# What Do Patients Know About Anesthesia and Anesthesiologists?

**DOI:** 10.3390/jcm14238459

**Published:** 2025-11-28

**Authors:** Dagmara Skowrońska, Aleksandra Garczyk, Anna Kluzik, Małgorzata Grześkowiak

**Affiliations:** 1 Poznan University of Medical Sciences, Department of Anesthesiology and Intensive Therapy Teaching, Święcickiego 6, 60-781 Poznan, Poland; akluzik@wp.pl (A.K.); mgrzesko@ump.edu.pl (M.G.); 2 University Clinical Hospital in Poznan, Poznan University of Medical Sciences, Grunwaldzka 55, 60-352 Poznan, Poland; 3 Multispecialist Municipal Hospital, Szwajcarska 3, 61-285 Poznan, Poland; garczykaleksandra@gmail.com; 4 Department of Anesthesiology, Intensive Therapy and Pain Treatment, Poznan University of Medical Sciences, Grunwaldzka 55, 60-352 Poznan, Poland

**Keywords:** anesthesia, perioperative care, health literacy, patient education, public health, anesthesiology

## Abstract

**Background/Objectives**: Nowadays, the importance of educating and ensuring communication with patients is also emphasized in groups of patients undergoing anesthesia. The safety and quality of services provided to this group of patients may be related to the information received by them. Therefore, the aim of this review is to explore the patients’ knowledge observed globally and discuss the potential influencing factors. **Methods**: This review was based on a search of PubMed using MeSH terms and keywords. Additionally, citation searching for relevant articles was performed. **Results**: The related literature illustrates high heterogeneity among studies with varying results. The knowledge concerning appropriate recognition of anesthesiologists as doctors ranged from 32,8% to 90.5%. However, most studies concluded that patients’ knowledge regarding anesthesia is poor. There was no homogenous pattern regarding the possible impact of age, sex, education, profession or previous anesthesia on patients’ knowledge. Patients’ most common concern was not waking up after anesthesia. The response to patients’ varying knowledge may be the use of educational aids including online alternatives. This approach limits the use of medical resources and may help to alleviate patients’ anxiety. **Conclusions**: Future studies may focus on a thorough analysis of knowledge in a representable population followed by an observation of aspects shaping the level of education. The precise influence of patients’ education on anesthesia outcomes is yet to be determined. However, further investigation may bring appropriate clinical guidance and help to ensure the best quality of anesthesia services is provided.

## 1. Introduction

It is believed that engaging patients in the healthcare system as partners plays an important role in safety maintenance [1]. Currently, the possibilities and requirements for patients’ safety improvement in anesthesia seem to be common across various countries [2]. According to the Helsinki Declaration on Patient Safety, it is relevant to engage and educate patients regarding their safety and its management [3]. Perceived quality of anesthesia seems to be another crucial issue. Information regarding general anesthesia that patients received was a frequent factor influencing their satisfaction [4]. A written information form handed out before the surgery was associated with a higher satisfaction level in a randomized–controlled trial [5]. It was also found to be enough to increase patients’ knowledge [5]. Besides satisfaction with the perioperative period, suffering from anxiety may be an important factor for both patients and medical personnel. Preoperative anxiety was estimated at 48% among the global patient population [6]. The higher level of anxiety in the preoperative period was associated with a greater need for emotional support [7]. Data regarding the influence of anxiety on the perioperative period is limited. However, the subgroup of patients after complex general surgery who complained of preoperative anxiety had a higher rate of postoperative complications [8]. Additionally, anxiety was associated with worse surgery outcomes and postoperative pain [9]. The level of anxiety was found to be lower when additional information was provided for patients before the surgery [10]. In contrast, another study showed no correlation between preoperative information and level of anxiety. However, the provided information was associated with better knowledge and a shorter time for interviewing a patient before surgery [11]. These findings may be crucial, as according to enhanced recovery after surgery (ERAS^®^) guidelines, education should be implemented as a step before admission to a hospital [12]. Such a strategy may help to alleviate levels of anxiety and should be used among others instead of routine pharmacologic anxiolysis [12].

The aim of this literature review is to evaluate data regarding patients’ knowledge about anesthesia. Furthermore, the clinical suggestions and directions for future studies will be discussed. We believe that assessing patients’ knowledge and perspectives regarding anesthesia is an important step in the matter of better clinical practice. It may also bring appropriate indications based on patients’ needs.

## 2. Materials and Methods

This review was based on a search of PubMed only, as it is preliminary to the following research regarding the state of knowledge in Poland. For the review, MeSH terms and keywords were applied, such as anesthesia, anesthesia conduction, anesthesia general, anesthesia local, analgesia, anesthetics, anesthesiology, sedation, postoperative care, postoperative pain, perioperative care, perioperative period, health literacy, informed consent, patient education as a topic, patient participation, knowledge, awareness, and education. Additionally, citation searching for relevant articles was performed. Two independent researchers assessed the results and determined their relevance by titles and abstracts. Applicable articles in English were included for further analysis. Articles regarding the population during hospitalization and the general population randomly selected for surveys were assessed. Furthermore, research regarding knowledge of the population irrespective of hospitalization time and with no outcomes about anesthesiologists, anesthesiology or anesthesia were excluded. Results were discussed and presented in a narrative form. Studies regarding hospitalization period are presented as a table (Table 1).

## 3. Results

This research work was based on a review of articles mainly from two areas: public knowledge about anesthesiologists and anesthesiology and knowledge about anesthesia.

A total of 29 articles from various continents and countries were reviewed. The variety of answers to the questions asked resulted from various factors, including gender, age, education and even the region of the country. A significant number of scientific papers demonstrate patients’ limited knowledge on the above topics [13,14,15,16,17,20,21,24,26,27,32,34,35,36,37].

Common awareness about anesthesiologists and anesthesiology was the first to be analyzed.

Many studies highlighted the insufficient knowledge of the respondents. Research conducted in Ethiopia showed that as many as two thirds of study participants had low knowledge about anesthesiologists and anesthesiology, and only 28.3% had a “good level of knowledge” [19]. In another study conducted in Northern Ghana, half of the patients did not know what an anesthesiologist is, and about a quarter of the respondents indicated that the anesthesiologist is an operating room nurse, which indicates a basic lack of education in this area [15].

In contrast, Italian research during the COVID 19 pandemic showed that 90.5% of patients had good knowledge of the role of an anesthesiologist [22]. A summary of the reviewed articles on the main outcome regarding anesthesiologists is presented in Table 1.

Further verification of awareness of particular anesthesiology- and anesthesia-related issues was attempted.

### 3.1. Knowledge About Anesthesiologists

First of all, an issue often raised in scientific papers is the question of patients’ knowledge of whether an anesthesiologist is a physician. Answers under this theme were at various levels. However, most researchers pointed to inadequate patient knowledge.

Respondents’ affirmative responses to questions regarding anesthesiologists being doctors ranged from 32.8% for New Delhi, India, to as high as 99% for Switzerland [38,39]. An interesting form of research was proposed by Brazilian scientists. They examined the state of knowledge before and after the operation. They showed an increase in patients’ knowledge after the procedure, and for the question of whether the anesthesiologist is a specialized doctor, positive results were obtained for 79.1% before and 87.4% after surgery [30]. The detailed results on this issue for particular articles are included in Table 1.

Awareness of the role of anesthesiologists also seems to be insufficient. According to a study conducted in a Nigerian hospital, half of the patients knew that an anesthesiologist is a qualified doctor, but 48.5% of the patients had no idea what their role is [14].

Similar results were obtained in Hispanic patients in California. It was reported that most of the respondents knew that anesthesiologists are specialized doctors (70% of respondents) and that they deal with “putting patients to sleep”. Nevertheless, patients did not associate the exact role of an anesthesiologist with the operating theater and during surgical procedures [21].

In most of the analyzed studies, patients also had little knowledge about who manages postoperative pain, takes care of them during surgery or takes part in resuscitation [16,18,36].

It was shown that two thirds of respondents in China believed that the role of an anesthesiologist during surgery is to relieve pain, while only 4.6% of patients from a Nigerian hospital reached the same conclusions [13,14]. Unsatisfactory results in terms of knowledge about the tasks of anesthesiologists were also obtained in other countries. In Saudi Arabia, almost 32% of respondents assumed that anesthesiologists only work in the operating theater, while in Pakistan as many as 72.3% incorrectly assessed the role of the anesthesiologist after surgery [18,35]. It is worth emphasizing that similarly low results were recorded in India, where only 27.1% of patients had good knowledge of the role of an anesthesiologist, and in Jordan only 15% [24,28]. Furthermore, only 19% of Caribbean country respondents and 17% from Minnesota (USA) presented knowledge about the work of anesthesiologists in the intensive care unit [33]. On the other hand, only 4% of Turkish respondents had this knowledge [32].

However, some studies show that respondents associated anesthesiologists mainly with “putting people to sleep” [14,17,21,36]. Patients also associate anesthesiologists with pain relief [30]. Occasionally, nevertheless, patients equate the role of the anesthesiologist with helping patients, being an aid to the surgeon or directing the surgeon’s actions [17].

Additionally, a low level of knowledge about the education process of anesthesiologists was also found. Despite the fact that three-quarters of the respondents in the Swiss experiment knew that the role of an anesthesiologist is not limited to the operating theater, only one-fifth of the patients correctly estimated the duration of postgraduate training, and 45% believed that the anesthesiology team works under the supervision of the surgeons [39]. Similarly, in studies performed in Portugal, the time required for education and training of anesthesiologists was generally underestimated, with the majority of patients (52.9%) reporting that they did not know the time required for training and education [31].

### 3.2. Knowledge About Anesthesia

Complementarily, another significant topic to consider is knowledge of anesthesia. The results in this regard also vary depending on the study. Only 26.7% of the surveyed respondents in India knew what anesthesia was [24]. Among respondents in Saudi Arabia (Jazan), up to 81.2% of respondents were knowledgeable about different types of anesthesia, while in Pakistan only 48.1% [18,35]. Respondents surveyed in another region of Saudi Arabia (Jeddah) were most familiar with local anesthesia (92.5%), with a similar level of knowledge about general anesthesia (89.9%) and the least knowledge about regional anesthesia (66.7%) [36]. However, the results in Turkey are slightly different and amount to 58.8% having knowledge about general anesthesia, 30.7% about local anesthesia, 12.8% about regional anesthesia and 30.7% not knowing about different types of anesthesia [32]. Knowledge in this topic was examined slightly differently in Ethiopia by asking what the different types of anesthesia were, and only 26% correct answers were obtained [20]. Patient preferences regarding the type of anesthesia were also examined and it was noted that general anesthesia was chosen more willingly [18,30]. In one study, the most common reason for preferring general anesthesia over regional anesthesia was that 12.98% of patients believe general anesthesia has no or fewer risks, and 10.82% of patients preferred general anesthesia due to fear of seeing something during surgery and refused regional anesthesia due to concerns about headaches and back pain. However, this proves that patients have insufficient knowledge about general anesthesia [18].

Knowledge about the induction of anesthesia is also relevant and the results are as follows: 83.3% of Italian patients know how to induce anesthesia, while 57.7% of Saudi patients responded correctly to the administration of anesthesia by intravenous or inhalation [16,22].

### 3.3. Fears Related to Anesthesia

Research on the Chinese population has shown that approximately 40% of patients have a neutral attitude to the risks associated with anesthesia and its addiction [13]. Meanwhile, almost 70% of patients in a study conducted in Northern Ghana were unaware of the possible risk of complications after anesthesia [15]. Very similar results were obtained in studies at a hospital in Ibadan. Almost 65% of patients did not know of any complications after anesthesia [14]. On the other hand, among the patients of the Saudi Arabian population (Jazan), 69% of them stated that anesthesia is safe and 31% that it is not [35], while as many as 48% of Irish patients are afraid of anesthesia [23,35]. Hispanic patients living in Los Angeles also fear devastating and rare complications (such as death under anesthesia, brain damage, paralysis) after procedures. Women were the most likely to express this fear. The top fear patients cited was fear of pain, followed by death and brain damage [21]. Similarly, in the Nigerian patient population, the fear of death and not waking up was the most frequently mentioned fear [14]. In a study conducted at Korle-Bu Teaching Hospital in Accra, Ghana, patients were mainly afraid of the surgical procedure itself (45.7%) as well as the pain associated with the surgery (28.9%) [17]. A study by Xue Wu et al. found that a significant number of patients are also convinced of the addictive potential and negative effects of anesthetic drugs on the organism [13]. Overall, fears of pain during or after surgery, not waking up after surgery, and waking up during surgery were dominant [15,21,22,25,32].

Studies conducted on Hispanic patients showed that patients with chronic illnesses, women, and those with higher knowledge scores had greater concerns about anesthesia [21].

According to a study conducted in Porto, Portugal, women were more afraid than men about not waking up after surgery, nausea and vomiting, and waking up during surgery at statistically significant levels. No statistically significant differences were found in terms of assessed fears between groups with and without prior anesthesia experience [31].

Furthermore, Italian researchers conducted a study of what type of anesthesia patients fear most. They obtained results that patients are most afraid of general anesthesia (42.5%) and spinal/epidural anesthesia (36.8%) compared to nerve block (9.8%), local anesthesia (7%) and the sedation procedure itself (3.9%) [22]. In addition, some studies also included questions about patients’ feelings, and one of them asked about the feeling of safety during anesthesia. Researchers showed that only about 1/3 of patients were not concerned about their safety due to anesthesia [19,21]. An attempt was also made to measure trust in the anesthesiologist and the mean trust score on a scale from 0 to 4 was 2.6 ± 1.2 (mean ± SD). The researchers indicate that trust was mainly influenced by the respondents’ knowledge about anesthesia [21].

### 3.4. Sources of Knowledge

In accordance with a study carried out in India, patients took their knowledge for the most part from the surgeon (about 65%) and not from the anesthesiologist [25]. The statistics presented in the survey conducted in China are quite different. The vast majority (nearly 82%) of the patients surveyed obtained their knowledge about anesthesia directly from the anesthesiologist, while only about 22% of those surveyed obtained their knowledge from the surgeon. The other most common sources of knowledge were the Internet and then relatives and friends [13]. Patients surveyed in Saudi Arabia gained their knowledge of anesthesia mainly from the media (36.8%) and from doctors (32.0%). The authors also indicated the source of knowledge to be school for 18.9% of the respondents, which may suggest the implementation of education in this field [34]. Also encouraging are results indicating that patients in Ghana (79.9%) have heard about anesthesia at a tertiary health care facility [17]. In another study, 81.1% of patients sought information about anesthesia from only one source, while 11.8% from more than one. The most common source of information was direct/indirect experience (56.3%) and physicians (26.9%), while other options accounted for less than 25% [22]. Surprisingly, the distribution of the dependence of the source of knowledge on the frequency of “medical advice” was presented by Bagabas et al. [36]. They found that sources of knowledge differed according to the frequency of hospital visits and the results were as follows: doctors were the most common source of information, especially for those who visit the hospital at least once a month, while social media and television were the sources of knowledge for most people who visit for medical advice at least once every six months.

Moreover, another important issue is the correlation of knowledge about anesthesiology and anesthesia with factors that may influence them, such as gender, age, education, profession or area of residence.

### 3.5. The Impact of Gender on Knowledge

Similarly, the results are divergent in the correlation of results with gender.

As an example, a study conducted in Saudi Arabia (Jazan) reports that sufficient knowledge about anesthesia was presented in 52.1% of men and 47.9% of women. However, a group with insufficient knowledge consisted of 70.5% men and 29.5% women (results presented are statistically significant) [35]. In contrast, researchers from the same country but a different localization, Jeddah, found that women source knowledge about anesthesia more often from the media and public awareness events than men at a statistically significant rate [36]. According to research in Godnar, Ethiopia, women have a greater knowledge of anesthesia and anesthesiologists, and “good knowledge” was reported by 40.6% of women, while in the group of men, this was only 27.4% [20]. In comparison, research conducted in Debre Tabor, north-central Ethiopia, showed that men were 1.9 times more aware of anesthesia and anesthesiologists than women [19]. In contrast, the results of a study in India presented no correlation between gender and knowledge about the preanesthesia check-up [26]. In addition, studies in Nigeria, the USA and Jordania found no statistically significant difference between gender and knowledge of the anesthesiologist’s role [14,28,29].

### 3.6. Impact of Age on Knowledge

In fact, there are papers indicating a correlation between age and knowledge of anesthesia.

For example, such a correlation was discovered by Xue Wu et al. [13]. Similar findings were also obtained during studies among Gazan residents in Saudi Arabia, where patients were divided into two age categories—over and under 30. Findings showed that among patients under 30 years of age, 62.7% showed sufficient knowledge of anesthesia and 51.9% did not, while in the group over 30 years of age, only 37.3% showed sufficient knowledge and 48.1% had no such knowledge [35]. Furthermore, another study discovered that as age increases, the level of knowledge about anesthesia decreases; however, the results did not achieve a statistically significant level [32]. In contrast, a Canadian study presented that those who were over 55 years of age were the most aware of anesthesiologists (in 51%), compared to those aged 35–54 (in 36%) and 18–34 (in 35%) [37]. According to one research paper, it was emphasized that age was the only variable influencing patients’ level of information about anesthesia, pointing out that patients over the age of 60 had more knowledge of the anesthesiologist’s role than younger patients [28]. However, several papers present a non-relation between gender in the level of knowledge about anesthesiology. Among others, such conclusions were reached by Djagbletey et al., who showed no correlation between age and mean knowledge of anesthesia and anesthetists [17]. Similarly, researchers from India found no correlation between age and knowledge of preanesthesia check-up [26]. However, this does not correspond to the results obtained by researchers from New Delhi, India, who found a significant relationship between age and knowledge about anesthesiologists and anesthesia [38].

### 3.7. Impact of Education Level on Knowledge

The association between education and knowledge about anesthesiologists and anesthesia has been frequently studied. Several papers have reported that there is no correlation between education level and knowledge of anesthesiologists. These include one study conducted in Canada, where the educational level of participants had no effect on their knowledge about a person administering general anesthesia [37]. Similarly, a study that divided respondents into groups with education completed at school and those with after-school education found no statistically significant differences [28]. Nagrampa et al. reached similar conclusions [21]. On the other hand, Eyelade et al. showed in surveys that respondents with a higher education had significantly greater knowledge about the role of an anesthesiologist compared to patients with secondary and primary education [14]. Similarly, Leite et al. found that correct answers regarding whether an anesthesiologist is a doctor were less frequent in people with a lower level of education [30]. Studies from the USA and the Caribbean showed a significant relationship between knowledge of anesthesiologists’ roles and education level [29,33]. Additionally, in a study conducted in West Bank, Palestine, results about knowledge of anesthesiologists were significantly better for those with higher education and a degree in medical/health sciences [27]. Moreover, respondents from a study conducted in New Delhi were divided into groups according to the completion of 10 classes, and it was proven that knowledge of anesthesiologists and anesthesiology is higher for those above 10 classes [38]. Associations were found between respondents’ education, not only for knowledge about anesthesiologists, but also for information about anesthesia. In Saudi Arabia, awareness that anesthesia was administered by a physician specializing in anesthesiology, that induction was intravenous, and that an anesthesiologist was caring for patients during surgery correlated well with a high level of education [16]. Very similar conclusions were reached by researchers from Saudi Arabia, Jazan, pointing to the influence of the level of education on the patient’s knowledge of anesthesiology and the role of anesthesiologists [35]. The level of education, compared to other factors such as previous experience with surgery, gender, and age, showed the greatest influence on the answers regarding anesthesiologists and anesthesia [24]. Also, in India, the percentage of correct answers regarding the preanesthesia examination depended significantly on the level of education. Illiterate patients and those with only primary education had significantly less knowledge compared to graduates and postgraduates [26]. Similarly, in Turkey, higher education was associated with better knowledge of anesthesia [32]. A study conducted in Ethiopia found no statistically significant difference between knowledge and education, while another study conducted in another part of Ethiopia, Debre Tabor, found statistical significance, reporting that the greatest knowledge was found in those with higher education [19,20].

### 3.8. Impact of Occupation on Knowledge

The impact of profession on knowledge of anesthesiology was also examined. Several studies mention that profession is one of the factors influencing a patient’s knowledge.

Based on research conducted in Ethiopia, it was shown that the profession performed had an impact on the knowledge of anesthesiology [19]. However, it is necessary to take into account the way in which professions were divided in this study—into only four professional groups (Government employee, Private business, Farmer, Other). The researchers also emphasized the fact that government employees have wider access to health knowledge—for example due to access to the Internet at work and the possibility of participating in various events in the health sector—and that anesthesiology is a new specialization in their country. Another report showed that patients performing physical labor had significantly lower knowledge scores [27]. An interesting analysis was carried out in Saudi Arabia, showing that higher income correlated with correct knowledge that an anesthesiologist is a physician administering anesthesia, that anesthesia is induced intravenously, and that the anesthesiologist provides appropriate care during the operation and will start resuscitation if necessary and continue postoperative pain relief [16]. On the other hand, a study conducted in New Delhi found no association between socioeconomic status and knowledge about anesthesiologists and anesthesiology [38]. Some of the reports also raise issues related to the performance of medical professions and their correlation with knowledge about anesthesia. Healthcare professionals or medical students had significantly better knowledge about anesthesia [13]. Similarly, in the case of knowledge about anesthesiologists, the percentage of people who had sufficient knowledge was higher among medical professionals than among non-medical professionals [35].

### 3.9. Impact of Place of Residence on Knowledge

Several studies have raised the issue of the influence of the inhabited area on patients’ knowledge. At the outset of their work, Indian researchers highlighted that more than 75 percent of the Indian population lives in rural areas, and knowledge regarding anesthesia and anesthesiologists is limited even among patients living in urban areas [25].

In a study conducted in Saudi Arabia, urban residents had a better knowledge of the role of the anesthesiologist and this was statistically significant compared to rural residents, who reflected poor awareness in this regard [16]. Ethiopian researchers came to similar conclusions, presenting how patients living in urban areas had 6.34 times more knowledge than those in rural areas [20]. In contrast, in Jordan, no statistically significant differences were found between urban or rural residence and knowledge about the role of an anesthesiologist [28]. Moreover, in the research work of Arefayne et al., based on previous reports, they emphasized that patients in developing countries have poorer knowledge compared to developed countries, and the reason for this may be a low level of education, and less access to information in the media and the Internet [19].

### 3.10. Impact of Other Factors

Some researchers have also examined the influence of religious beliefs on knowledge about anesthesiologists. In a research study carried out in one of the Nigerian hospitals, there was no evidence of the influence of faith (the respondents were Muslims or Christians) on the knowledge about the role of an anesthesiologist [14]. In addition, researchers in India conducted pre- and post-anesthesia (follow up) surveys and noted a significant improvement in responses during post-operative follow-up. They explain this by providing comprehensive care before anesthesia [24].

## 4. Discussion

Most papers reported insufficient patient knowledge about anesthesiologists and anesthesia. Many factors such as age, gender, and education may affect this knowledge. However, patients’ awareness seemed to depend not only on their individual characteristics, but also on external factors such as previous surgeries or preoperative visits. In fact, a few studies show that prior exposure to anesthesia increases knowledge about anesthesiologists and the anesthesia procedure [13,16,18,19,20,29]. However, there are also findings that even with previous experience with surgery, patients may still have insufficient knowledge about anesthesia [17,28,31,39]. This points to the need for thorough explanation of anesthesia in pre-operative visits, even if the patient has had surgery before. The observed differences between studies may be related to the disparities in general education and income level between countries and due to in-population inequality.

Several studies have shown that preoperative visits have an impact on reducing anxiety before surgery and that low patient knowledge was due to a lack of explanation of anesthesia procedures by anesthesiologists [13,14,15,25,35]. The fact that knowledge about anesthesia reduces anxiety before procedures and thus influences the results of surgical procedures was emphasized [32]. Patients would like to receive a wide range of information during the preoperative visit, including all possible complications. Moreover, it has been proven that introducing the anesthesiologist into the preoperative clinic and informing the patient about anesthesia influenced patients’ self-confidence and encouraged them to talk about their health, concerns and possible complications of the surgery [23,24,29]. In addition, lack of knowledge often leads to increased anxiety and therefore it is essential to increase patient knowledge [13]. This is consistent with the ERAS protocol, which notes the importance of involving the patient in the treatment process and talking to the patient in reducing patient anxiety. The ERAS protocol highlights the importance of providing full information about anesthesia in premedication, as talking to the patient may have a similar effect in minimizing preoperative anxiety as long-acting sedative drugs. The patient’s attitude is also significantly influenced by trust in medical workers, while the main influence on trust in the anesthesiologist is the patient’s knowledge about anesthesia [13,21].

This also proves the need to spend time with the patient, explaining the procedures to improve the patient–doctor relationship [21]. The authors of this article propose that this role may be played by anesthesiologists, but other trained medical personnel (e.g., interns, final-year medical students, anesthetic nurses) may also attempt to provide advice. Visits to preoperative clinics are time-limited and include not only patient education but primarily examination of the patient and their qualification for the procedure. This makes it difficult to accurately and in detail explain all procedures performed on patients. An additional proposed solution is to provide patients with information brochures about anesthesia during registration at pre-operative clinics, which patients believe could be helpful [23,29]. However, this is not consistent with research conducted in Switzerland, which showed that additional information materials did not improve patients’ knowledge [39]. Therefore, the effectiveness and usefulness of such brochures should be considered individually for each healthcare entity.

In addition, it is worth emphasizing the need to propagate knowledge about anesthesia and possible complications in private practices such as dental or aesthetic medicine clinics. It has been shown that the tremendous increase in demand for aesthetic medicine and also plastic surgery is having an impact on the development of anesthesia in private practices, and this is the fastest growing segment of anesthesiology practice [40].

The other point that should be raised is the impact of superstitious beliefs and incorrect information that patients believe in on their fear of surgery. Some studies indicate increased anxiety and anxiety among superstitious patients associated with having surgery on Friday the 13th [41]. Furthermore, patients are also concerned about general anesthesia, especially with regard to possible brain damage, death and intraoperative awareness, as well as the disclosure of personal information involuntarily during anesthesia [42]. Moreover, some patient fears exist regarding the failure of surgery depending on the phase of the moon, or the horoscope and zodiac sign [43,44,45]. Notably, superstitious beliefs have also been observed among medical professionals [43]. This all the more so demonstrates the need to promote anesthesia knowledge and dispel myths, especially since numerous studies have shown that increased anxiety on the part of the doctor or patient during surgery may lead to poorer outcomes [41].

Additionally, it seems relevant to find an appropriate method of providing information about anesthesia to the elderly, who are a challenging population for anesthesiologists nowadays [46,47]. Aging itself is a factor influencing dosing of anesthetics [48] and the observed body changes associated with aging are influencing perioperative periods [49], which might be relevant information during patients’ education. Additionally, the observed cognitive impairment among elderly patients may influence the outcomes and postoperative period [50] and should be considered as an influencing factor for understandings of the performed procedures.

The fact that a significant number of procedures require general or regional anesthesia and the person primarily responsible for patient monitoring and safety is the anesthesiologist is worth highlighting. The anesthesiologist is the physician responsible for managing surgery-related complications such as bleeding, septic shock and life-threatening conditions in the ICU during anesthesia (in several countries, anesthesiology is a combined specialty with intensive care). Thus, it is all the more important to establish a good patient relationship and cooperation at every stage of surgery, including during the pre-operative visit, during which most information about the patient may be obtained and the patient’s concerns explained. This could affect the operation, the occurrence and treatment of complications. Nowadays, the mortality caused by anesthesia has decreased with the help of the Anesthesia Patient Safety Foundation, which is also a source of knowledge for patients and physicians with regards to the safety of the perioperative period [51].

The limitations of the present work should also be discussed. Firstly, we based our search on PubMed only, which may bias the results. The results of the works analyzed vary from each other, which may be influenced by conducting research at different times, by asking some other questions, other characteristics of the respondents surveyed, varying research samples, and dissimilar ways of measuring the regularity of knowledge. Although research was conducted in many countries, most of them were carried out only in one or a few hospitals, which only reflected the awareness of patients in a limited area. Therefore, this may make it challenging to generalize the results obtained. A possible solution to the problem of the variety of surveys that are conducted would perhaps be the introduction of a validated questionnaire, translated into several languages and distributed through online channels. Nevertheless, it is important to collect information about patients’ knowledge and to search for solutions based on this knowledge to affect patient education.

## 5. Conclusions

Most of the analyzed works indicate poor knowledge about anesthesiologists and anesthesia. It is advisable to disseminate general knowledge in this field. Furthermore, standardized tests should be introduced and carried out on a larger scale. In addition, knowledge obtained from statistics could indicate which groups have insufficient knowledge and which should be particularly targeted with educational activities.

## Figures and Tables

**Table 1:** Studies on knowledge about anesthesia in populations of hospitalized patients.

Study	Country	Cases	Anesthesiologist as a Doctor	Anesthesiologist Responsible for Anesthesia	Main Outcome
Wu X et al. [13]	China	429	N/A	62.7%—induce anesthesia, relieve pain	Age, being a healthcare professional or medical student and previous anesthesia were related with better awareness
Eyelade OR et al. [14]	Nigeria	229	50%	23.8%—induce anesthesia, monitor vital signs, relief pain; 17.7%—induce anesthesia	Poor knowledge regarding anesthesia
Suglo S. et al. [15]	Northern Ghana	100	N/A	31%—induce sleep; 49%—qualified to give anesthetics	Poor knowledge regarding anesthesia
Baaj J. et al. [16]	Saudi Arabia	170	55.3%	N/A	Socioeconomic status correlated with opinion regarding anesthesia
Djagbletey R. et al. [17]	Ghana	279	85.1%—trained doctor or nurses	45.5%	Most of patients recognized role of anesthesiologist in the operating room, but not outside of it
Kadri I. et al. [18]	Pakistan	231	51.5%	N/A	Poor knowledge regarding anesthesia
Arefayne NR. et al. [19]	Ethiopia	307	28.3%—assessed as having ‘a good knowledge’		~2/3 patients had poor knowledge regarding anesthesia
Fentie Y. et al. [20]	Ethiopia	367	N/A	71.5%	Sex, residency, education, and previous anesthesia were associated with level of knowledge
Nagrampa D. et al. [21]	USA	300	70%	82%	Poor knowledge regarding anesthesia
D’Agostino F. et al. [22]	Italy	1400	90.5%	44.1%—induce anesthesia, monitor vital signs	Good knowledge during COVID-19 pandemic
Smith A. et al. [23]	Ireland	100	78%	N/A	Most patients recognized role of anesthetist only in operating room, not outside of it
Ray A.K. et al. [24]	India	361	54.6%	27.1%—induce anesthesia, monitor vital signs	Poor knowledge regarding anesthesia
Singh P.M. et al. [25]	India	214	N/A	N/A	Poor knowledge regarding anesthesia
Singla D. et al. [26]	India	1000	N/A	N/A	Poor knowledge regarding anesthesia
Zahran A. et al. [27]	Palestine	411	78.6%	79.1%	Poor knowledge regarding anesthesia and anesthesiologist outside the operating room
Bataineh A.M. et al. [28]	Jordan	505	37%	75%	Poor knowledge regarding details of anesthesia
Garcia-Marcinkiewicz A.G. et al. [29]	USA	502	86%	N/A	Poor knowledge regarding anesthesia despite high education level of a population
Leite F. et al. [30]	Brazil	518	79.1%—before surgery; 87.4%—after surgery	35.5%—before surgery; 43.5%—after surgery	After surgery significantly more patients recognized that anesthesiologist is a physician
Ribeiro C.S. et al. [31]	Portugal	204	66.2%	N/A	Poor knowledge regarding anesthesia
Sagün A. et al. [32]	Turkey	250	73.6%	60.4%	Poor knowledge regarding anesthesia
Hariharan S. et al. [33]	Caribbean	371	59%	N/A	Education status correlated with knowledge about anesthesiologist as a doctor

## Abbreviations

The following abbreviations are used in this manuscript:

ERAS	enhanced recovery after surgery
ICU	Intensive Care Unit

## References

[1] World Health Organization (2021). Global Patient Safety Action Plan 2021–2030: Towards Eliminating Avoidable Harm in Health Care.

[2] Warner M.A., Arnal D., Cole D.J., Hammoud R., Haylock-Loor C., Ibarra P., Joshi M., Khan F.A., Lebedinskii K.M., Mellin-Olsen J. (2022). Anesthesia Patient Safety: Next Steps to Improve Worldwide Perioperative Safety by 2030. Anesth. Analg..

[3] Mellin-Olsen J., Staender S., Whitaker D.K., Smith A.F. (2010). The Helsinki Declaration on Patient Safety in Anaesthesiology. Eur. J. Anaesthesiol. EJA.

[4] Hawkins R.J., Swanson B., Kremer M.J. (2012). An Integrative Review of Factors Related to Patient Satisfaction with General Anesthesia Care. AORN J..

[5] Straessle R., Gilliard N., Frascarolo P., Rossat J., Albrecht E. (2011). Is a Pre-Anaesthetic Information Form Really Useful? Usefulness of a Pre-Anaesthetic Information Form. Acta Anaesthesiol. Scand..

[6] Abate S.M., Chekol Y.A., Basu B. (2020). Global Prevalence and Determinants of Preoperative Anxiety among Surgical Patients: A Systematic Review and Meta-Analysis. Int. J. Surg. Open.

[7] Salzmann S., Rienmüller S., Kampmann S., Euteneuer F., Rüsch D. (2021). Preoperative Anxiety and Its Association with Patients’ Desire for Support—An Observational Study in Adults. BMC Anesth..

[8] Kassahun W.T., Mehdorn M., Wagner T.C., Babel J., Danker H., Gockel I. (2022). The Effect of Preoperative Patient-Reported Anxiety on Morbidity and Mortality Outcomes in Patients Undergoing Major General Surgery. Sci. Rep..

[9] Levett D.Z.H., Grimmett C. (2019). Psychological Factors, Prehabilitation and Surgical Outcomes: Evidence and Future Directions. Anaesthesia.

[10] Bondy L.R., Sims N., Schroeder D.R., Offord K.P., Narr B.J. (1999). The Effect of Anesthetic Patient Education on Preoperative Patient Anxiety. Reg. Anesth. Pain Med..

[11] Kakinuma A., Nagatani H., Otake H., Mizuno J., Nakata Y. (2011). The Effects of Short Interactive Animation Video Information on Preanesthetic Anxiety, Knowledge, and Interview Time: A Randomized Controlled Trial. Anesth. Analg..

[12] Jankowski C.J. (2017). Preparing the Patient for Enhanced Recovery After Surgery. Int. Anesthesiol. Clin..

[13] Wu X., Li H., Li X., Yang Y. (2024). Knowledge, Attitude, and Practice of Non-Emergency Surgical Patients toward Anesthesia. Sci. Rep..

[14] Eyelade O., Akinyemi J., Adewole I. (2014). Patients’ Perception and Knowledge of Anaesthesia and Anaesthetists—A Questionnaire Survey. South. Afr. J. Anaesth. Analg..

[15] Suglo S., Gross J., Boakye Yiadom A., Wuni A., Achaab S. (2020). Assessing Patients’ Knowledge on Anesthesia Services at Tamale Teaching Hospital. SAGE Open Nurs..

[16] Baaj J., Takrouri M.S.M., Hussein B.M., Al Ayyaf H. (2006). Saudi Patients’ Knowledge and Attitude toward Anesthesia and Anesthesiologists—A Prospective Cross-Sectional Interview Questionnaire. Middle East J. Anaesthesiol..

[17] Djagbletey R., Aryee G., Essuman R., Ganu V., Owusu-Darkwa E., Owoo C., Ahiaku F., Yawson A.E. (2017). Patients’ Knowledge and Perception of Anaesthesia and the Anaesthetist at a Tertiary Health Care Facility in Ghana. South. Afr. J. Anaesth. Analg..

[18] Kadri I. (2015). Awareness of Patients Regarding Anesthesia; Attitude towards Basic Types of Anesthesia Techniques. Prof. Med. J..

[19] Arefayne N.R., Getahun A.B., Melkie T.B., Endalew N.S., Nigatu Y.A. (2022). Patients’ Knowledge and Perception of Anesthesia and the Anesthetists: Cross-Sectional Study. Ann. Med. Surg..

[20] Fentie Y., Simegnew T. (2021). Awareness and Its Associated Factors towards Anesthesia and Anesthetists’ among Elective Surgical Patients in Debre Tabor Comprehensive Specialized Hospital, North Central Ethiopia 2021: Cross-Sectional Study. Ann. Med. Surg..

[21] Nagrampa D., Bazargan-Hejazi S., Neelakanta G., Mojtahedzadeh M., Law A., Miller M. (2015). A Survey of Anesthesiologists’ Role, Trust in Anesthesiologists, and Knowledge and Fears about Anesthesia among Predominantly Hispanic Patients from an Inner-City County Preoperative Anesthesia Clinic. J. Clin. Anesth..

[22] D’Agostino F., Fusco P., Sammartini E., Sinagoga A., Poloni J., Angeletti S., Fabris S., Ferri C., Pelosi P., Agrò F. (2022). Patients’ Knowledge of Anesthetic Practice and the Role of Anesthetists during COVID-19 Pandemic. J. Anesth. Clin. Res..

[23] Smith A., Mannion S. (2013). Irish Patients Knowledge and Perception of Anaesthesia. Ir. Med. J..

[24] Ray A.K., Mondal D., Porel S., Chattopadhyay A. (2024). A Study of Patient’s Knowledge and Perception about Anaesthesia and the Anaesthesiologist in a Tertiary Care Hospital in Kolkata. Int. J. Acad. Med. Pharm..

[25] Singh P.M., Kumar A., Trikha A. (2013). Rural Perspective about Anesthesia and Anesthesiologist: A Cross-Sectional Study. J. Anaesthesiol. Clin. Pharmacol..

[26] Singla D., Mangla M. (2015). Patient’s Knowledge and Perception of Preanesthesia Check-up in Rural India. Anesth. Essays Res..

[27] Zahran A., Besharieh F., Hamdan Y., Alsadder T., Jaber M., Shawahna R. (2024). Knowledge and Perceptions of the Roles of Anesthesiologists as Providers of Healthcare Services: Toward Better-Educated Patients. BMC Health Serv. Res..

[28] Bataineh A.M., Qudaisat I.Y., El-Radaideh K., Alzoubi R.A., Abu-Shehab M.I. (2020). Patients’ Perception of the Practice of Anaesthesia in a Teaching Hospital in Northern Jordan: A Survey. BMC Anesthesiol..

[29] Garcia-Marcinkiewicz A.G., Long T.R., Danielson D.R., Rose S.H. (2014). Health Literacy and Anesthesia: Patients’ Knowledge of Anesthesiologist Roles and Information Desired in the Preoperative Visit. J. Clin. Anesth..

[30] Leite F., da Silva L.M., Biancolin S.E., Dias A., Castiglia Y.M.M. (2011). Patient Perceptions about Anesthesia and Anesthesiologists before and after Surgical Procedures. Sao Paulo Med. J..

[31] Ribeiro C.S., de Barros Mourão J.I. (2015). Anesthesiologist: The Patient’s Perception. Braz. J. Anesthesiol. (Engl. Ed.).

[32] Sagün A., Birbiçer H., Yapici G. (2013). Patients’, Who Applied to the Anesthesia Clinic, Perceptions and Knowledge about Anesthesia in Türkiye. Saudi J. Anaesth..

[33] Hariharan S., Merritt-Charles L., Chen D. (2006). Patient Perception of the Role of Anesthesiologists: A Perspective from the Caribbean. J. Clin. Anesth..

[34] Geddawy M.A., Alkraydees S.S., Almadhi M., Alashqar S.A., Alghelfes A.I., Aljabaan B. (2023). Public Awareness and Knowledge About Anesthesiology and the Role of Anesthesiologists in Al-Qassim Province, Saudi Arabia. Cureus.

[35] Mohajer A.A., Matiri A.Y., Jaafari A.A., Almalki N.A., Alfaqih A.M., Gosada A.H., Sanba H.A., Khamis K.A., Shayani R.T. (2018). A Survey on Jazan Public Awareness about The Role of The Anesthesiologists. Egypt. J. Hosp. Med..

[36] Bagabas A.M., Ashi M.M., Alamoudi A.O., Alaidarous S.A., Filemban S.K., Bahaziq W.K. (2017). Knowledge about Anesthesia and the Role of Anesthesiologists among Jeddah Citizens. Int. J. Res. Med. Sci..

[37] Neilipovitz D., Cooke-Lauder J., Bryson G.L., McIsaac D.I. (2023). Canadian Public Perception of Anesthesiologists: Results from a National Survey. Can. J. Anesth./J. Can. Anesth..

[38] Singh T., Sharma S., Banerjee B., Garg S. (2018). Knowledge Regarding Anesthesiologist and Anesthesiology among Patients and Attendants Attending a Rural Hospital of New Delhi. J. Educ. Health Promot..

[39] Kindler C.H., Harms C., Alber C. (2002). The patients’ perception of the anaesthetist in a Swiss university hospital. Anaesthesist.

[40] Jovanovic K., Kalezic N., Sipetic Grujicic S., Zivaljevic V., Jovanovic M., Savic M., Trailovic R., Vjestica Mrdak M., Novovic M., Marinkovic J. (2022). Patients’ Fears and Perceptions Associated with Anesthesia. Medicina.

[41] Ranganathan S., Riveros C., Geng M., Chang C., Tsugawa Y., Ravi B., Melchiode Z., Hu S., Kobashi K., Miles B.J. (2024). Superstition in Surgery: A Population-Based Cohort Study to Assess the Association Between Surgery on Friday the 13th and Postoperative Outcomes. Ann. Surg. Open.

[42] Roublah E.A., Alqurashi R.N., Kandil M.A., Neama S.H., Roublah F.A., Arab A.A., Boker A.M. (2020). Patients’ Concerns and Perceptions of Anesthesia-Associated Risks at University Hospital: A Cross-Sectional Study. Saudi J. Anaesth..

[43] Schuld J., Slotta J.E., Schuld S., Kollmar O., Schilling M.K., Richter S. (2011). Popular Belief Meets Surgical Reality: Impact of Lunar Phases, Friday the 13th and Zodiac Signs on Emergency Operations and Intraoperative Blood Loss. World J. Surg..

[44] Faschinger E.-M., Vécsei-Marlovits P.V., Rabensteiner D.F., Weingessel B. (2017). The Influence of Lunar Phases on Complications in Cataract Surgery: An Analysis of 16,965 Patients. J. Ophthalmol..

[45] May M., Braun K.-P., Helke C., Richter W., Vogler H., Hoschke B., Siegsmund M. (2007). Lunar Phases and Zodiac Signs Do Not Influence Quality of Radical Cystectomy—A Statistical Analysis of 452 Patients with Invasive Bladder Cancer. Int. Urol. Nephrol..

[46] United Nations Department of Economic and Social Affairs (2020). World Population Ageing 2019.

[47] Bouwhuis A., van den Brom C.E., Loer S.A., Bulte C.S.E. (2021). Frailty as a Growing Challenge for Anesthesiologists—Results of a Dutch National Survey. BMC Anesthesiol..

[48] Akhtar S. (2021). Neurological Aging and Pharmacological Management of Geriatric Patients. Curr. Anesthesiol. Rep..

[49] Strøm C., Rasmussen L.S., Steinmetz J. (2016). Practical Management of Anaesthesia in the Elderly. Drugs Aging.

[50] Chen L., Au E., Saripella A., Kapoor P., Yan E., Wong J., Tang-Wai D.F., Gold D., Riazi S., Suen C. (2022). Postoperative Outcomes in Older Surgical Patients with Preoperative Cognitive Impairment: A Systematic Review and Meta-Analysis. J. Clin. Anesth..

[51] https://www.apsf.org/about-apsf/apsf-history/.

